# Hydrogen peroxide fogging of isolation gowns: Disinfection efficacy and VOC emissions

**DOI:** 10.1371/journal.pone.0338332

**Published:** 2025-12-08

**Authors:** Shay Iyer, Zenhwa Ouyang, Arathi Vinayak

**Affiliations:** 1 Department of Surgical Oncology, VCA West Coast Specialty and Emergency Animal Hospital, Fountain Valley, California, United States of America; 2 Independent Researcher, Ontario, Canada; Satyawati College, University of Delhi, INDIA

## Abstract

Isolation gowns constitute the majority of the PPE waste generated daily worldwide. Disinfection with reuse that is easily reproducible without the need for expensive equipment or trained personnel is needed. This study evaluated the use of 9% hydrogen peroxide fogging using a commercially available fogger on isolation gowns heavily inoculated in the torso region of the gown with *Staphylococcus epidermidis* and *Escherichia coli* to challenge efficacy. Cultures were obtained on isolation gowns immediately after inoculation from both the interior as well as the exterior of the gowns before disinfection. Gowns were then fogged with 9% hydrogen peroxide (HP) for 1 min and 30sec. A four-hour trying time was instituted, and gowns were cultured from the torso region again. All cultures were incubated at 37 °C for 72 hours. CFU at 48- and 72-hours post-incubation were obtained. Safety of this methodology was studied by evaluating Total Volatile Organic Compounds (TVOCs) as a VOC index (1–500) as well as in parts per million (PPM) generated during HP fogging of gowns, aiming to determine whether the use of vacuum-aeration (VA) led to an improvement in VOCs generated. Results indicate that fogging with HP was effective at reducing *S. epidermidis* and *E. coli* as measured by CFU (100% reduction at 72 hours for both organisms). VOC index and PPM were greater without VA than with VA. The maximum Δ(Index_BC_) (191) and Δ (PPM_BC_) (0.301) occurred at 90 minutes and then fell to between 156 and 187 for Δ (Index_BC_) and 0.202 and 0.253 for Δ (PPM_BC_). This corresponded to 82% (156/191) and 98% (187/191) of the maximum VOC index and 67% (0.202/0.301) and 84% (0.253/0.301) of the maximum PPM without VA. Our results indicate that HP fogging may be a viable option for disinfection and reuse of single use isolation gowns and VA may be a valuable additional step in improving air quality during the process.

## Introduction

Medical waste is a burgeoning problem worldwide, with a staggering 1.6 million tons of personal protective equipment (PPE) generated daily [1]. Isolation gowns manufactured from non-degradable materials are a standard part of the PPE attire worn by healthcare workers to reduce cross-transmission, prevent transmission of healthcare-associated infections (HAI), as well as reduce the risk of infection acquisition by hospital personnel. Disinfection and reuse of PPE has been a topic explored by numerous publications since the COVID pandemic [2–4]. Given that isolation gowns are the largest percentage of PPE waste by weight (85%), it is imperative to explore options that enable safe reuse [5].

Hydrogen peroxide is a widely used disinfectant/sterilant in the form of an aqueous solution, vapor, or aerosol [6]. It is an ideal “biocide,” defined as an agent that is easy to store, safe to use, has a long-lasting effect, is environmentally friendly, and is chemically compatible [7]. It is widely used in healthcare due to its excellent antimicrobial activity, with efficacy against highly resistant endospores and viruses [8]. The most common organisms encountered in hospital settings are *Staphylococcus aureus* (30.61%), followed by *Pseudomonas aeruginosa* (16.33%) and *Escherichia coli* (14.29%) in terms of HAIs [9]. From 2013 and 2017, in a tertiary care center, *E. coli* predominated in the outpatient setting (44%) and *S. aureus* and *E. coli* predominated in the inpatient setting, 21% and 24% respectively [10]. In addition, another cause of IV catheter or prosthetic infections is *Staphylococcus epidermidis* [11]. Personnel wearing isolation gowns can thus serve as vectors of contamination, and the pathogens could pose a risk to the personnel as well. Thus, if gown reuse is to be considered, disinfection must be tested against the more common pathogens to ensure re-use is safe not only for the patients but also for the personnel donning the reused gowns.

Hydrogen peroxide is used in disinfection due to its effectiveness, and its breakdown into water and oxygen makes it eco-friendly. Superior material compatibility, availability in various forms, and non-carcinogenic properties are additional reasons it has been explored in a variety of disinfection/sterilization applications in recent times. According to the CDC, most EPA-registered disinfectants, such as hydrogen peroxide, have labeled contact times of 10 minutes. A 7% hydrogen peroxide solution is fungicidal in 5 minutes, virucidal at 5 minutes, and bactericidal at 3 minutes of contact time [12,13].

Hydrogen peroxide fogging has become a leading fumigation system as it is less hazardous to personnel, has a broad spectrum of activity, and non-toxic breakdown products [14]. Fumigation can be delivered either as a vapor or as an aerosol. Vapors are generated from minimum concentrations of 30–35% HP and require a heat source, while aerosols are generated from concentrations of 5–12% HP [14]. The more common vapor systems are vaporized HP (VHP), HP vapor (HPV), and hydrogen peroxide gas plasma (HPGP) used for equipment sterilization. Fogging systems supply HP as an aerosol. Aerosolized HP (aHP) is a fine mist of 5–10% hydrogen peroxide emitted through a nozzle, producing smaller particles <10um that can disinfect items. The standard method involves a pressure generator that produces small aerosols with <50PPM silver cations [15]. The two agents work together to increase microbial kill [16]. The average time for sterilization is 2–3 hours [17]. A more recent study using a novel aHP method containing plasma-activated HP (no silver cations) showed consistent inactivation of bacterial growth of *Geobacillus stearothermophilus* [18]. aHP is generally used in the fumigation of rooms with equipment present [14,19,20]. A review article compiling published literature of aHP, VHP, and HPGP has succinctly summarized each of these procedures [17].

Nearly all studies regarding aHP, VHP, HPV, and HPGP are on non-isolation gown PPE equipment or hard, non-porous surfaces. To the authors’ knowledge, there are no studies on isolation gowns inoculated with organisms and evaluated using any of the HP methods. A study on VHP on coverall body suits was disinfected for reuse using 7–8% HP and showed no damage to the coveralls, physical deformity, or damage fabric architecture of the melt blown layer on electron microscopy [2]. While biologic indicators were tested (*E. coli, M. smegmatis, B. stearothermophilus*), the coveralls themselves were not inoculated with bacteria. This study suggests that HP could be used for disinfection of isolation gowns made of similar melt-blown fabrics, such as isolation gowns, without compromising integrity. Isolation gowns are mostly manufactured using non-degradable synthetic materials like polypropylene, spunbound-meltdown-spunbound (SMS), or polyethylene-coated non-woven material [21].

Volatile organic compounds (VOC) describe a group of organic compounds in vapor form [22]. There are methods to differentiate very volatile organic compounds (VVOC) from semi-volatile organic compounds (SVOC), but the sum of the concentration of all VOCs is a TVOC value, which is the total volatile organic compounds [22]. TVOCs arise from various sources, ranging from contaminants in atmospheric air, decomposition, chemical reactions, commercial/industrial processes, etc. Recently, VOCs from both the disinfectants themselves as well as the release of products from chemical interactions of disinfectants with the materials they are in contact were published as an emerging concern in medical practice [23,24]. In the study by Ou et al, the composition of PPE masks (polypropylene and polyethylene) released different VOCs following disinfection with sodium hypochlorite and ultraviolet germicidal irradiation, with the former being a safer option for mask disinfection [24].

TVOC sensors on the market each measure the TVOC values in different parameters, making direct comparisons challenging. SPG30 sensor detects hazardous and toxic gases using the TVOC parameter in PPM [25]. The SPG40 and BME688 sensors use an IAQ-index out of 500 (unitless) [25,26]. The newer sensor is the SGP41 (used in this study), which evaluates the TVOC as a VOC index from 1–500, representing from best to worst quality range [26]. When the SGP41 sensor was tested against 10 different VOC solvents at different volumes. This sensor outperformed the BME688 for 9/10 tested VOCs, and the threshold that should be selected to sound the alarm for the SPG41 was recommended as 200 in the VOC index [26]. Overall, when the authors compared the SGP41 against SGP30, SGP40, and BME680, the SGP41 had the best response to most VOCs, and this sensor has been recommended as the better choice for air pollution monitoring devices [26].

For this report, hydrogen peroxide fogging (HPF), more similar to aHP, refers to a method where 9% HP mist with particle sizes between 15–25 microns is generated using a fogger to disinfect isolation gowns. HPF in a 12% concentration, and 500 ppm hypochlorous acid fog (HCF) utilizing the same methodology was evaluated against ethylene oxide (EO) sterilization in a recent study on isolation gowns, where percent CFU reduction was similar to ethylene oxide for both HPF and HCF in an uncontaminated gown scenario [27]. HPF vacuum-aeration (HPF-VA) is defined as HPF that is followed by VA to purge HP residues from the gowns after HP fogging. The goal of this study is to evaluate low-concentration 9% hydrogen peroxide disinfection of isolation gowns using hydrogen peroxide fogging (HPF) inoculated with *Staphylococcus epidermidis* and *Escherichia coli*. We hypothesized that HPF would result in a reduction of microbial burden in both *S. epidermidis* and *E. coli*. Our second hypothesis was that this reduction would be greater in *E. coli* compared to *S. epidermidis,* as the catalase activity for *Staphylococcus* species can lead to premature breakdown of HP. Our third hypothesis is that the TVOC trends will be lower over the first 4 hours after fogging when HPF-VA is used, when compared to HPF, remaining below a VOC index threshold of 200.

Development of eco-friendly, safe, inexpensive methods such as HPF and HPF-VA could prove to be worthy techniques to aid in disinfection and safe re-use of isolation gowns, especially in resource-limited and/or pandemic settings. HPF and HPF-VA could eliminate the need for dedicated disinfection areas and trained personnel, thereby reducing process costs and eliminating implementation-related barriers. Disinfection using these techniques would enhance pandemic preparedness.

## Materials and methods--methodology

### 9% Hydrogen peroxide fogging—*Staphylococcus epidermidis* (Item # 155556, Carolina Biological Supply Company, Burlington, North Carolina, United States)

**Fig 1:**
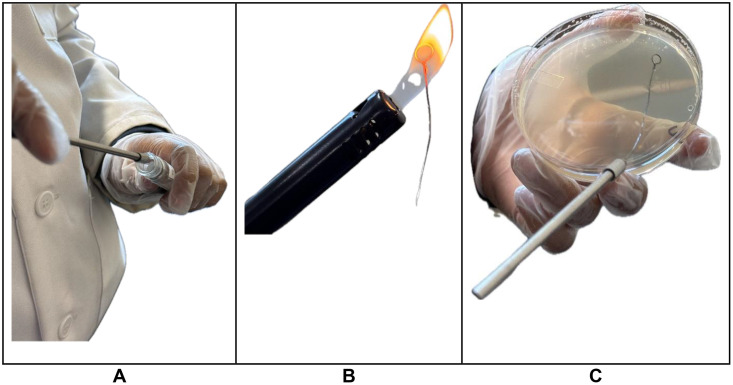
Streak plate technique. [A] Bacterium collected via a sterile loop from an agar slant in preparation for transfer. [B] The sterile loop was sterilized using a flame between each quadrant during streak plating. [C] A streak plate technique was used to transfer the bacterium from the agar slant to a nutrient agar plate.

In Group A, 9% hydrogen peroxide (HP) fogging was tested against *S. epidermidis* inoculated on 15 gowns. Agar slant bacterium was cultured onto 3 nutrient agar plates using the streak plate technique for 48 hours at 37 °C to cultivate a pure culture of colonies for inoculations (Fig 1).

**Fig 2:**
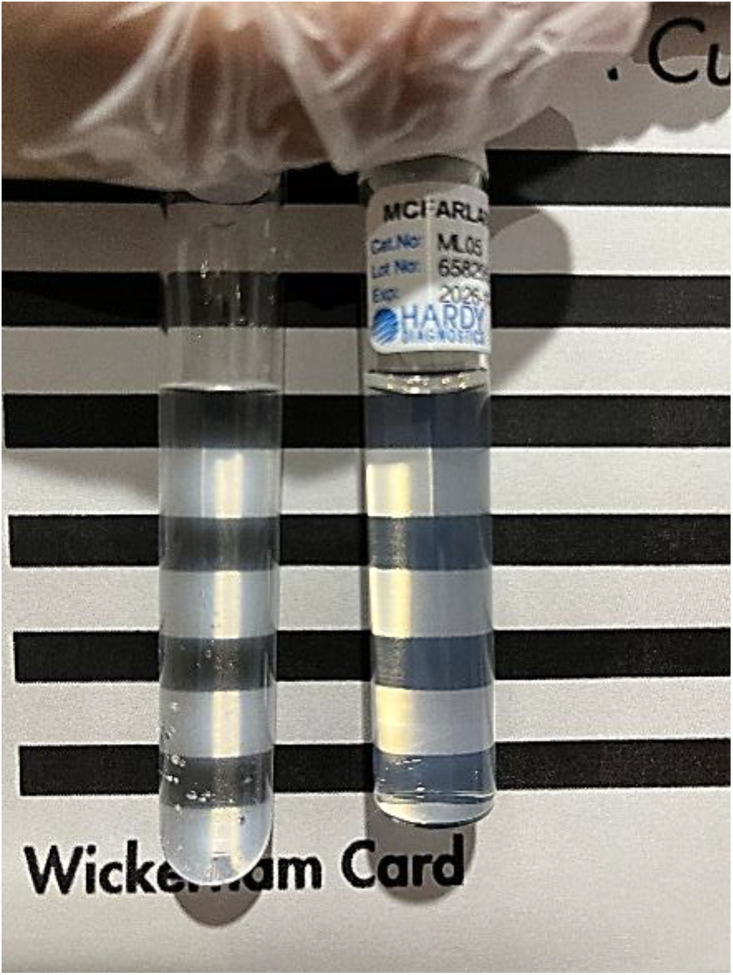
McFarland turbidity inoculum preparation. Inoculum preparation was performed using a 0.5 McFarland turbidity against a Wickerham card.

Colonies from these plates were suspended in sterile saline in an ethylene oxide-sterilized glass test tube, and a 0.5 McFarland turbidity standard suspension was prepared for the isolate using a Wickerham card (Fig 2).

**Fig 3:**
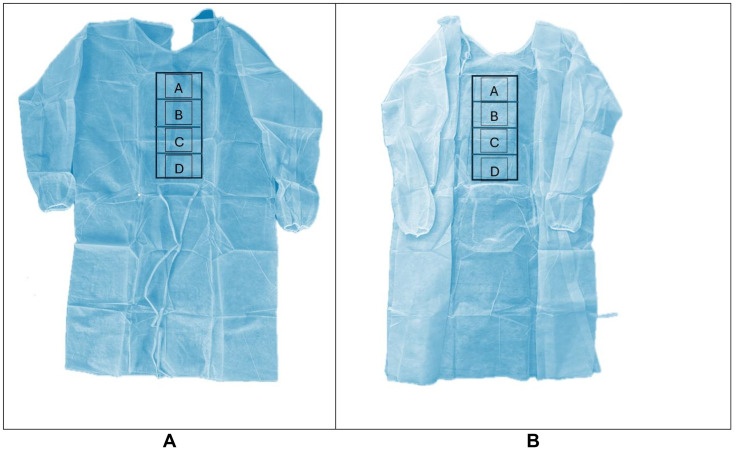
Four quadrants on the torso of isolation gowns are used for inoculum distribution and cultures. Isolation gown were marked with a 12 x 5 inch rectangle in the torso of the gown, divided into 4 equal quadrants for cultures. [A] Exterior of the gown with the represented area. [B] Interior of the gown with the represented area.

Our previous pilot study showed that the microbial burden on new isolation gowns out of the packaging is high, as the gowns are clean but not sterile [27]. Thus, ethylene oxide sterilization was performed on all gowns before inoculations to eliminate organisms present on the gowns at the onset. Isolation gowns before EO sterilization were premarked with a 12 in x 5 in rectangle in the torso region (Fig 3). This rectangle was further divided into 4 equal areas. The McFarland’s equivalent inoculum suspension was used to inoculate the gowns using a one sterile cotton-tipped applicator with a wooden stick end dipped into the suspension (0.139 ml per swab of inoculum) [28] and using one half of the swab for the first quadrant using a side-to-side streaking motion, and then the other side of the swab for the second quadrant. A second cotton tip applicator was then used in a similar fashion for quadrants three and four, all in a side-to-side streaking motion.

**Fig 4:**
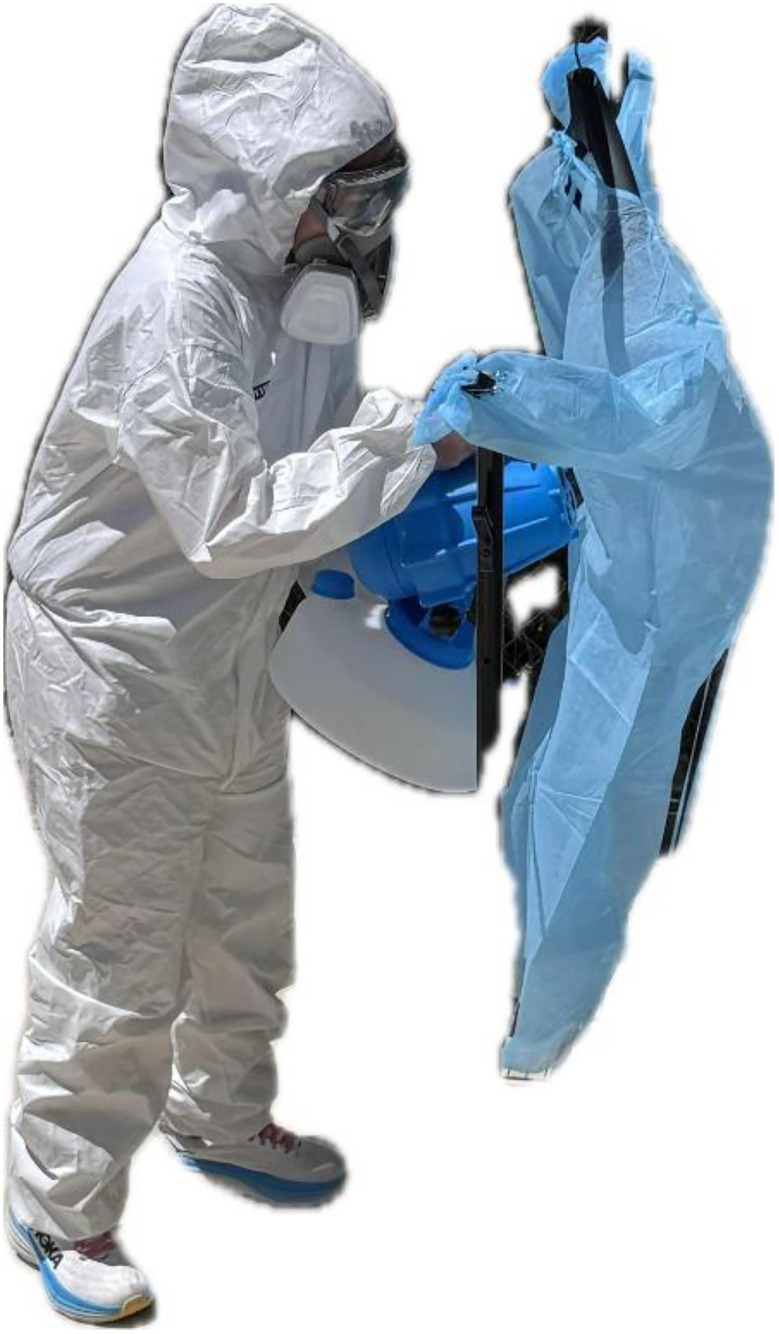
Hydrogen peroxide fogging is performed with a fogger. Both the exterior and interior of the gowns were fogged for a standardized 1 min:30sec total timed duration.

PPE consisting of sterile gloves, masks were used to prevent contamination. 10 min timed drying time/contact time was given for all 15 gowns after inoculation. A pre-disinfection culture was obtained using a contact plate of Tryptic soy agar (TSA) with Tween 80 and lecithin in two areas (quadrants A and C, Fig 3) in the designated area on the outside of the gown and the mirror areas on the inside of the gown on a single plate (15 predisinfection plates for 15 gowns). After donning coveralls, respirator, gloves, goggles, and shoe covers, 15 inoculated gowns were then fogged outdoors (Ceed4U, China) in the shade for 1 min:30 sec with 9% HP (diluted from 12%, SimpleNature, Fort Wayne, Indiana, United States); timed 45 seconds to fog the outside of the gown and 45 seconds to fog the back and inside of the gown (Fig 4).

The entirety of the gown was fogged, simulating real life when contamination of the gowns and their location may not be known. A drying time of 4 hours was instituted for all gowns. The gowns were then cultured in the two designated areas (quadrants B and D, Fig 3) using a second TSA contact agar plate with Tween 80 and lecithin on the outside and inside of the gowns on one plate (15 post-disinfection plates). Thus, there were a total of 15 contact plates prior to disinfection, and 15 contact plates after disinfection for the 15 gowns used in this group. All plates were then incubated at 37 °C for 72 hours with CFU counts performed by 2 readers at 48 and 72 hours and morphology to ensure any residual colonies were consistent with *Staphylococcus* colonies.

### 9% Hydrogen peroxide fogging—C600 *Escherichia coli* (Item # 124605, Carolina Biological Supply Company, Burlington, North Carolina, United States)

In Group B, the methodology was identical to Group A, except for the use of *E. coli* instead of *S. epidermidis*. Pre- and post-disinfection plates were incubated at 37 °C for 72 hours with CFU counts performed by 2 readers at 48 and 72 hours and morphology to ensure any residual colonies were consistent with *E. coli* colonies. Again, there were a total of 15 contact plates prior to disinfection, and 15 contact plates after disinfection for the 15 gowns in this group.

### Volatile organic compounds (VOC index and VOC PPM) for HPF

An SGP41 sensor (Sensirion SGP41 TVOC/NOX sensor, Sensirion AG, Stäfa, Switzerland) was used to record air quality post-fogging using VOC index values (1–500 unitless) and VOC in PPM of an isolated room of 1280 cubic feet, maintained at a constant temperature of 22.2 °C and humidity of 60%. Baseline VOC index values as well as VOC in PPM values were recorded before the onset of fogging. The VOC index and VOC in PPM represent values for *total* volatile organic compounds generated (i.e., TVOCs). 5 gowns were fogged using standard HPF as described in the previous section without inoculation. All 5 gowns were then placed together in the room with the air quality sensor, with the door to the chamber closed, and VOC index, as well as VOC PPM, were recorded every 30 minutes remotely using an Air Gradient Dashboard linked to the sensor. The last values recorded were at 4 hours post-fogging at the end of the timed drying post-fogging.

### Volatile organic compounds (VOC index and VOC PPM) for HPF-VA

The same SGP41 sensor was used to record air quality post-fogging after vacuum-assisted aeration using VOC index values (1–500 unitless) and VOC in PPM. The sensor was placed in an aerated room to get baseline values. 5 gowns were then fogged in the manner described above. Immediately following fogging, the 5 gowns were placed together in a vacuum chamber (PB Motor Tech 5-gallon Vacuum Chamber with 4.5CFM vacuum pump, China), and aeration was performed at 5 pa of vacuum for 30 minutes. The gowns were moved following vacuum aeration into the 1280 cubic feet room at 22.2 °C and 60% humidity with the sensor. Values were recorded remotely every 30 minutes until 4 hours post-fogging at the end of the timed drying post-fogging.

## Materials and methods—statistical analysis

Two readers (reader 1 and reader 2) evaluated the number of CFUs on each plate. Two approaches were used to consolidate the CFU of the readers. In the first approach, the intraclass correlation coefficient (ICC) between readers 1 and 2 was calculated for each time point. CFUs from the two readers were averaged if the ICC was 0.9 or more. For convenience, this approach was called “CFU consolidation approach 1”.

In the second approach, when disagreement occurred at pre-disinfection, the smaller count was used. When disagreements occurred at post-disinfection, the larger count was used. This ensured the most conservative estimate of the percent reduction in CFU. For convenience, this approach was called “CFU consolidation approach 2”. If the ICC in CFU consolidation approach 1 was below 0.9, only CFU consolidation approach 2 was used.

Plates with CFU exceeding 200 were recorded as 200 + . To ensure the most conservative estimate of the percent reduction in CFU, values exceeding 200 were truncated at 200. Percent reduction in CFUs was calculated according to the following equation:


$$\%\;reduction=\;\frac{CFU\;at\;\text{4 8}\;(or\;\text{7 2})hours\;predisinfection-CFU\;at\;\text{4 8}\;(or\;\text{7 2})\;hours\;postdisinfection}{CFU\;at\;\text{4 8}\;(or\;\text{7 2})hours\;predisinfection}*\text{1 0 0}$$

Statistical analyses were performed in R version 4.5.1. It was decided, a priori, to use Friedman tests (using base R) to understand whether significant differences existed among post-disinfection timepoints, and Mann-Whitney U tests (using base R) to detect significant differences in percent reduction between *E. coli* and *S. epidermidis* at each time point. If no variability existed across observed values, results were presented descriptively. Illustrations of the changes in TVOC over time with and without VA were provided for visual assessment and summary. If CFU and percent reductions were perfectly consistent across all plates for a timepoint, interrater agreement was not calculated, and only raw CFU and percent reductions were reported.

Longitudinal depictions of the VOC index, PPM, and changes in index and PPM were provided. Both VOC index and PPM absolute measurements were provided to allow for comparisons with previous literature where measurements were reported in either unit but not both. Changes (or deltas Δ) in index and PPM from values at time 0 were calculated to account for differences in baseline values (baseline-corrected values). Differences in baseline-corrected values without vacuum aeration and with vacuum aeration were calculated for each time point. For convenience, this will be called Δ (Index_BC_) and Δ (PPM_BC_).

## Results

**Fig 5:**
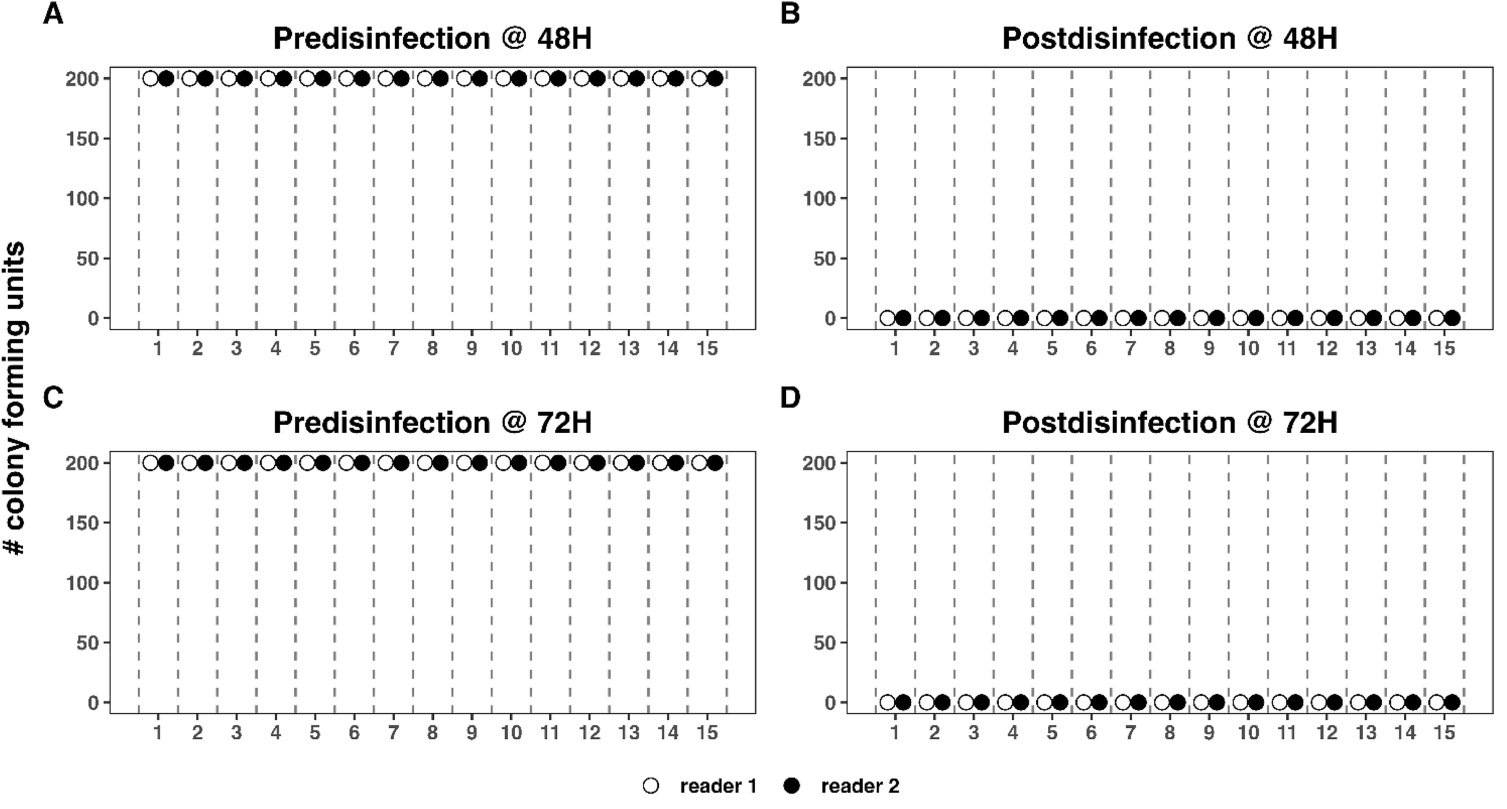
Reader CFU for *E. coli* at 48- and 72-hours pre- and post-disinfection for both observers. Reader CFU for *E. coli* at [A] 48 hours pre-disinfection; [B] 72 hours post-disinfection; [C] 48 hours pre-disinfection; and [D] 72 hours post-disinfection. Complete agreement occurs between readers for all plates at all timepoints. The open circle is Reader 1 (SI), and the black colored circle is Reader 2 (AV).

CFU of *E. coli* for each reader can be seen in Fig 5. Both readers recorded 200 CFUs at 48 and 72H pre-disinfection (Fig 5A and 5C, respectively) and 0 CFUs at 48 and 72H post-disinfection (Fig 5B and 5D, respectively).

**Fig 6:**
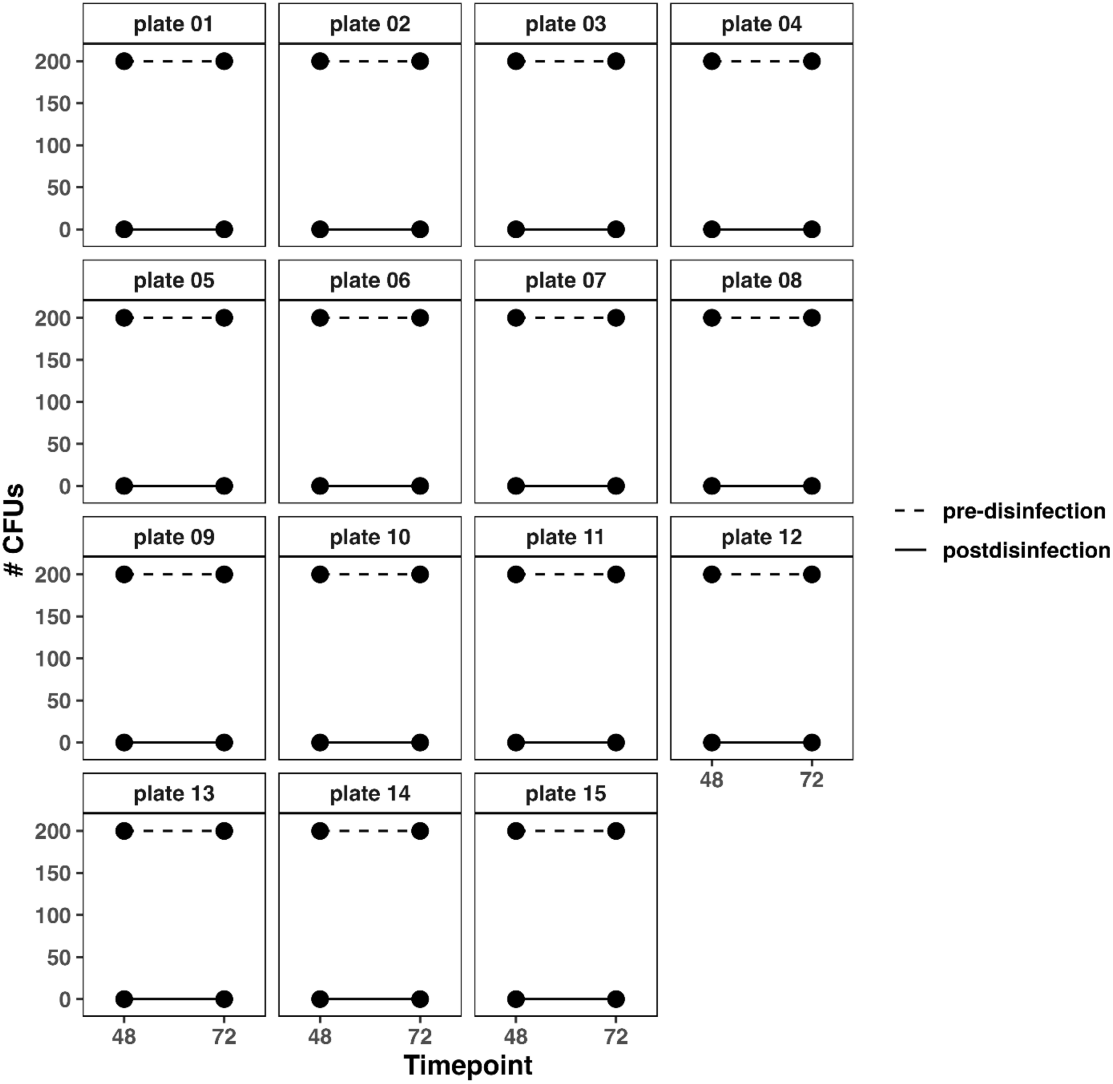
CFU counts at 48-and 72-hours pre- and post-disinfection for *E. coli.* Changes in CFU at each time point (48H and 72H) for *E. coli* pre-disinfection (dashed line) and post-disinfection (solid line).

**Fig 7:**
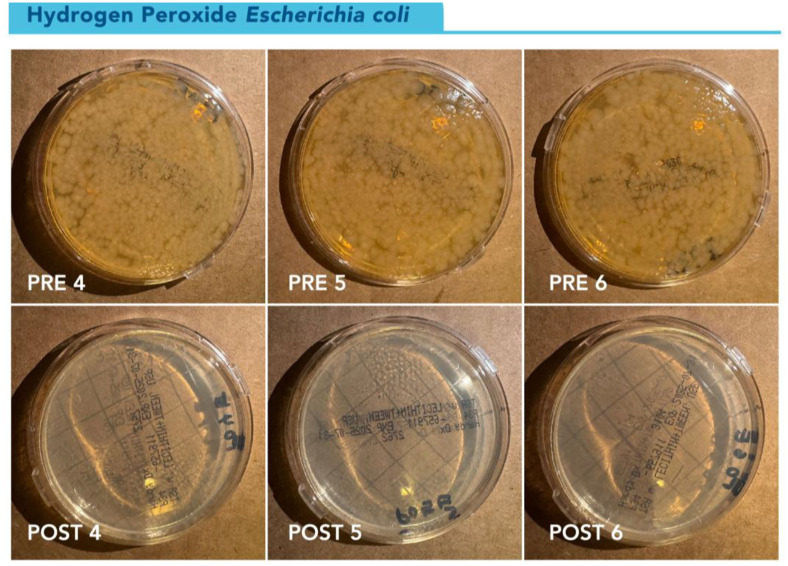
Visual representation of *E. coli* growth on TSA contact plates at 72 hours pre-and post-disinfection. TSA contact plates showing pre-disinfection growth of *E. coli* at 72 hours on the top row and post-disinfection cultures at 72 hours in the bottom row, showing 100% reduction of bacterial growth. Cultures from gowns 4, 5, and 6 are shown in the image.

Longitudinal changes in CFU counts over time can be seen in Fig 6. Percent reductions at 48H and 72H were 100% for all plates (Fig 7).

**Fig 8:**
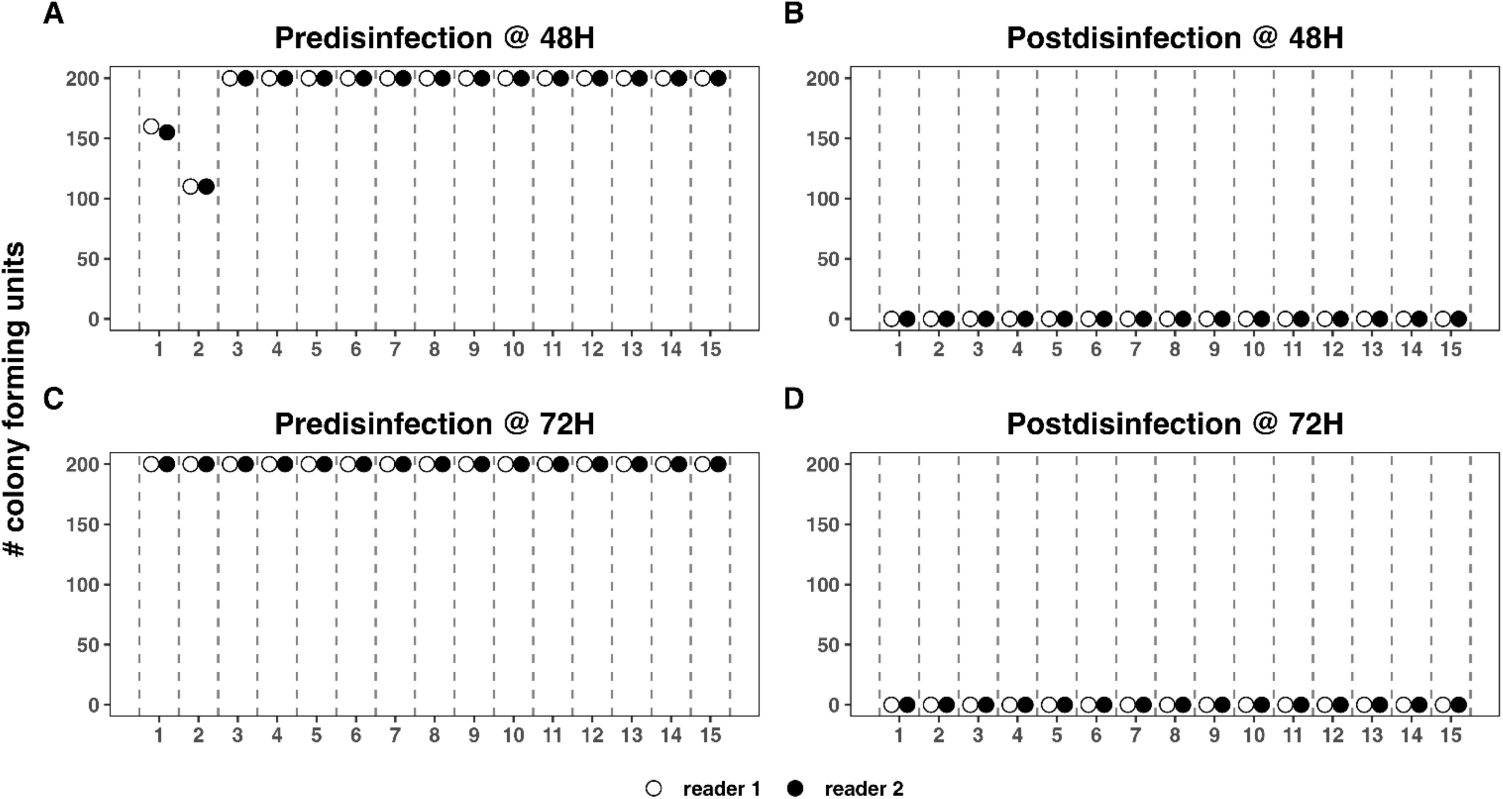
Reader CFU for *S. epidermidis* at 48 and 72 hours. At 48 hours pre-disinfection (A), reader agreement occurred for all plates except plate 1 (160 vs. 155 CFUs). Readers agreed on 110 CFUS for plate 2. For the remaining plates, readers agreed on 200 CFUs. At 48 hours post-disinfection (B), readers agreed on zero CFUs for all plates. At 72 hours pre-disinfection (C), readers agreed on 200 CFUs for all plates. And at 72 hours post-disinfection (D), readers agreed on 0 CFUs for all plates.

CFU of *S. epidermidis* for each reader can be seen in Fig 8. For *S. epidermidis*, at 48H pre-disinfection, readers 1 and 2 recorded 160 and 155 CFUs on plate 1, respectively (Fig 8A). On plate 2, both readers recorded 110 CFUs. Both readers recorded 200 CFUs on plates 3–15. The ICC at 48H pre-disinfection was 9.9x10^-5^ (95%CI: −0.5–0.5). Thus, the CFU consolidation approach 2 was used. Thus, the final CFU on plates 1 and 2 were 160 and 110. Both readers recorded 200 CFUs for all plates at 72H pre-disinfection (Fig 8C). Both readers recorded 0 CFUs for all plates at 48 (Figs 8B) and 72H (Fig 8D). The intraclass correlation coefficient was undefined at 72H pre-disinfection and 48 and 72H post-disinfection due to perfect agreement between readers and constant CFU counts across all plates. Since perfect agreement existed between readers, the CFU counts of a single reader (reader 1) were used as the final count for these plates.

**Fig 9:**
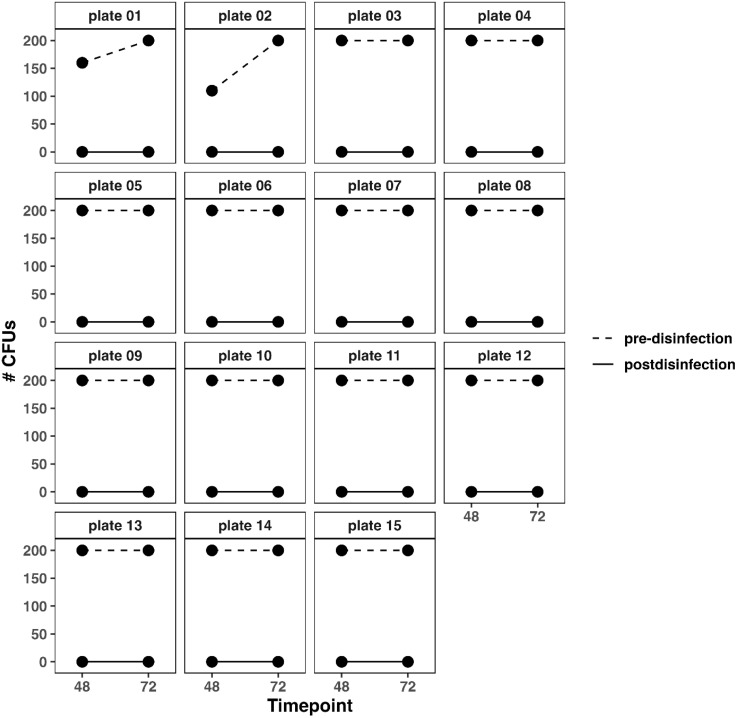
CFU counts at 48-and 72-hours pre- and post-disinfection for *S. epidermidis.* Changes in CFU counts at each time point (48H and 72H) for *S. epidermidis* pre-disinfection (dashed line) and post-disinfection (solid line).

**Fig 10:**
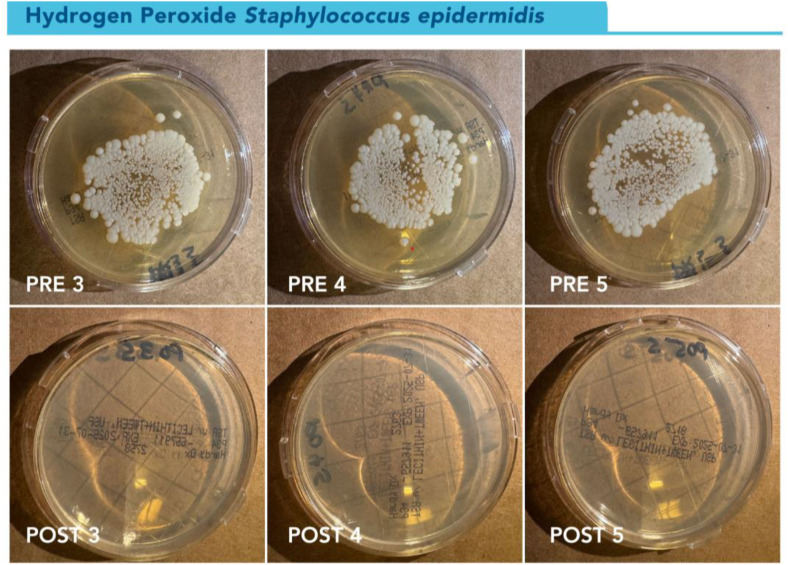
Visual representation of *S. epidermidis* growth on TSA contact plates at 72 hours pre-and post-disinfection. TSA contact plates showing pre-disinfection growth of *S. epidermidis* at 72 hours on the top row and post-disinfection cultures at 72 hours in the bottom row, showing 100% reduction of bacterial growth. Cultures from gowns 3, 4, and 5 are shown in the image.

Longitudinal changes in CFU counts over time can be seen in Fig 9. Percent reductions at 48H and 72H were 100% for all plates (Fig 10).

It was initially planned to assess significant differences in the percent reduction of CFU across time points. However, the observed percent reductions were consistently 0% across all time points, indicating a complete absence of CFU growth. As a result, there was no variability to permit statistical testing.

**Fig 11:**
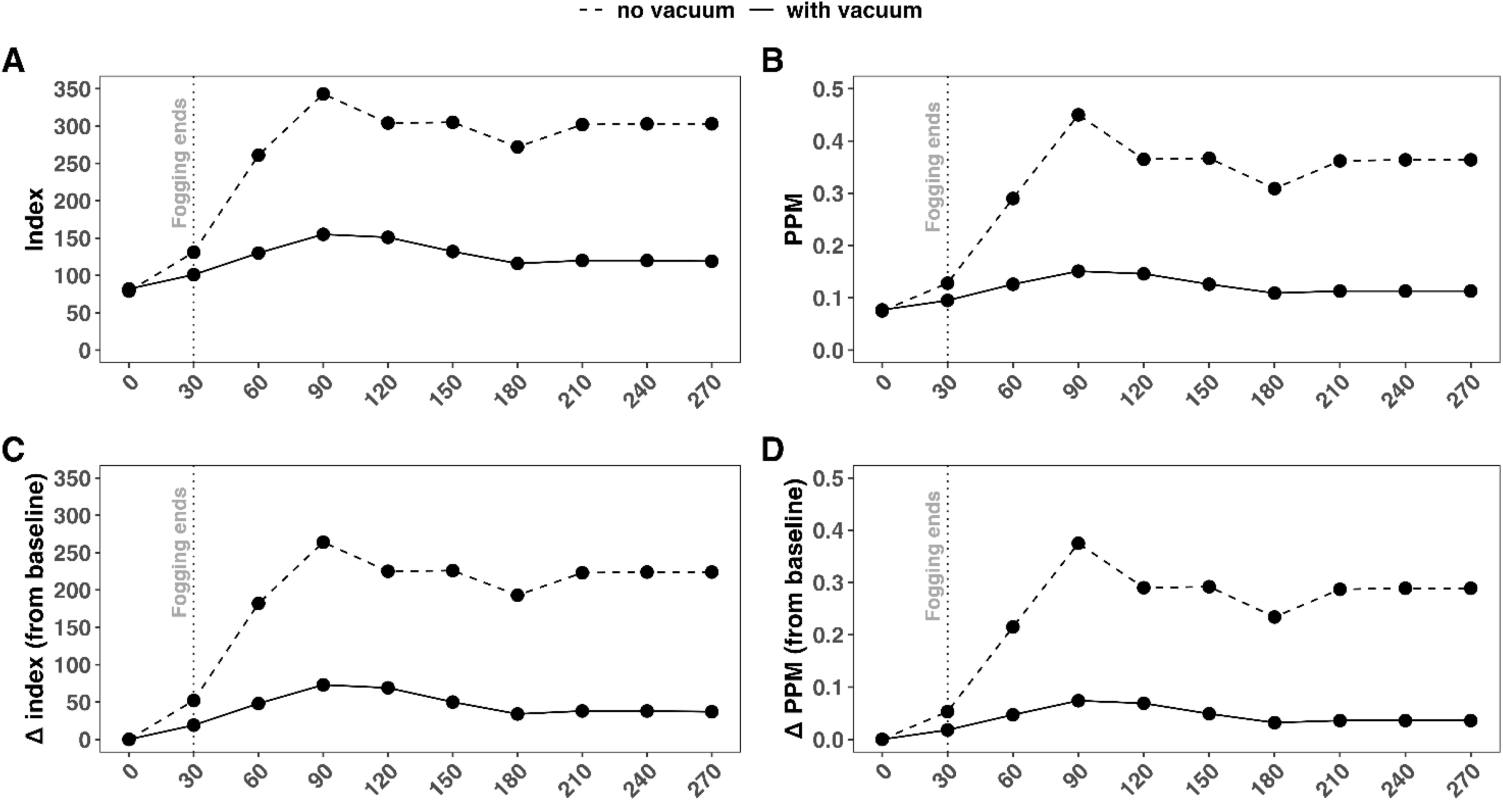
TVOC measurements with and without vacuum aeration following hydrogen peroxide fogging. Longitudinal measurements of TVOC over time with and without vacuum aeration: A) Index-based measurements; B) PPM-based measurements; C) changes in index-based measurements for baseline (time 0); and D) changes in PPM measurements for baseline.

TVOC are shown in Fig 11. At time 0, without vacuum aeration, the VOC index and PPM were 79 (Fig 11A) and 0.075 (Fig 11B), respectively. Without vacuum aeration, the maximum index and PPM achieved were 343 (Fig 11A) and 0.45 (Fig 11B), respectively, at 90 minutes (i.e., 60 minutes after fogging). This resulted in maximum increases in index and PPM from baseline of 264 (Fig 11C) and 0.375 (Fig 11D). By the end of the study, without vacuum aeration, the index and PPM dropped to 303 (Fig 11A) and 0.364 (Fig 11B), respectively. This resulted in increases in index and PPM from baseline of 224 (Fig 11C) and 0.289 (Fig 11D), respectively. At time 0 with VA, the VOC index and PPM were 82 (Fig 11A) and 0.077 (Fig 11B), respectively. With vacuum aeration, the maximum index and PPM achieved were 155 (Fig 11A) and 0.151 (Fig 11B), respectively, at 90 minutes (i.e., 60 minutes after fogging). This resulted in maximum increases in index and PPM from baseline of 73 (Fig 11C) and 0.074 (Fig 11D). By the end of the study, with vacuum aeration, the index and PPM dropped to 119 (Fig 11A) and 0.113 (Fig 11B), respectively. This resulted in increases in index and PPM from baseline of 37 (Fig 11C) and 0.036 (Fig 11D), respectively.

**Fig 12:**
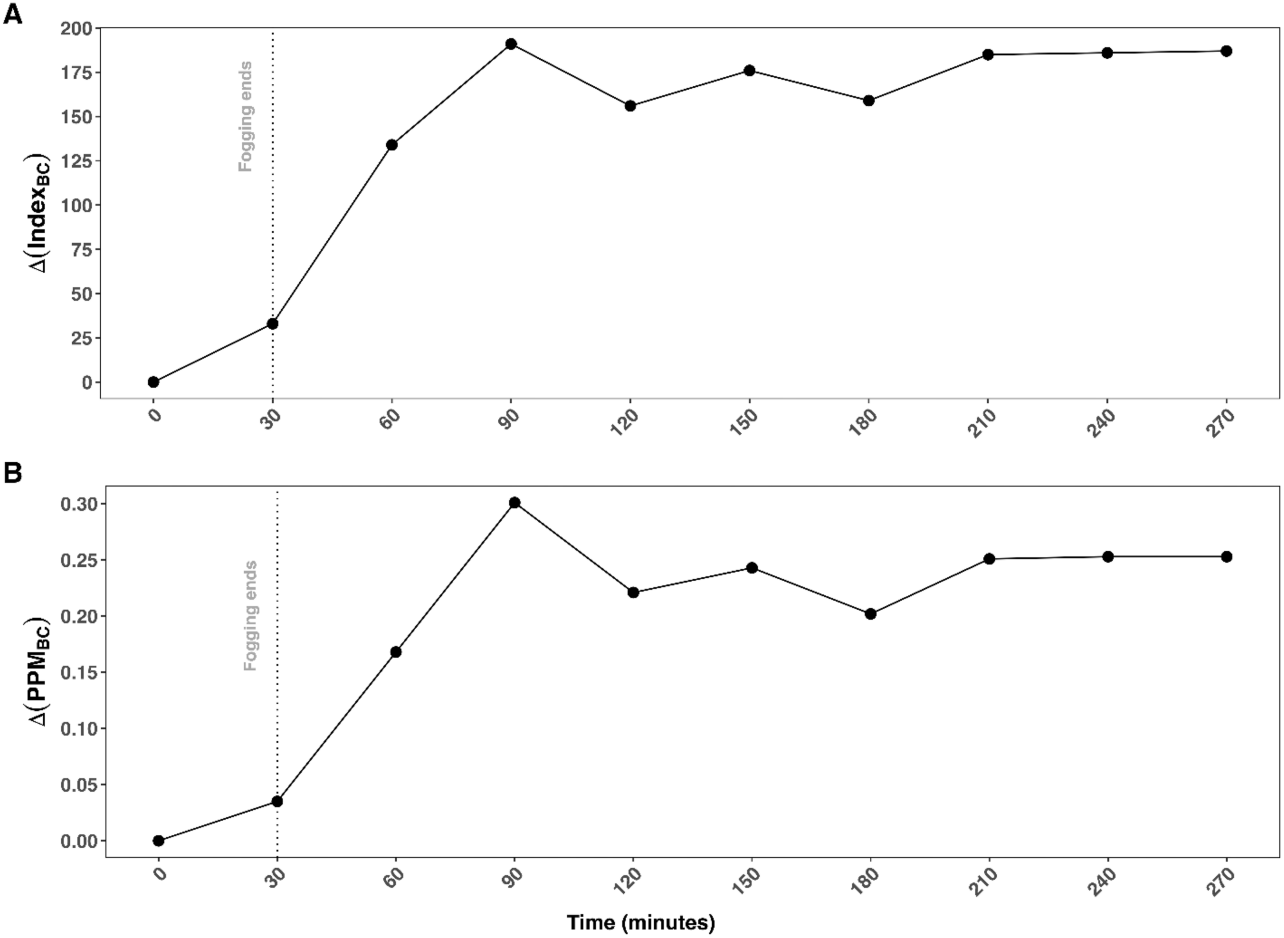
Baseline-corrected VOC index and PPM with and without vacuum-aeration. Differences in baseline-corrected VOC index (Δ(Index_BC_)) (A) and PPM (Δ(PPM_BC_)) (B) between fogging without vacuum and fogging with VA across timepoints.

At all time points, VOC index (Fig 12A) and PPM (Fig 12B) were greater without VA than with VA. The maximum Δ(Index_BC_) (191) and Δ(PPM_BC_) (0.301) occurred at 90 minutes and then fell to between 156 and 187 for Δ (Index_BC_) and 0.202 and 0.253 for Δ (PPM_BC_). This corresponded to 82% (156/191) and 98% (187/191) of the maximum VOC index and 67% (0.202/0.301) and 84% (0.253/0.301) of the maximum PPM without VA.

## Discussion

Our study evaluated the efficacy of HPF against *S. epidermidis* and *E. coli*. We elected to use inexpensive equipment and disinfectant in a methodology that is easily replicable in medical practices without access to VHP/HPV, HPGP, EO, or other more expensive sterilization equipment and dedicated personnel. This study provides proof-of-concept that HPF is effective at reducing the number of *E. coli* and *S. epidermidis,* as evidenced by a reduction in CFUs. The results thus supported the first hypothesis that HPF would be effective in eliminating a heavy inoculation of the two organisms. It did not support the second hypothesis, where we postulated that HPF would be more effective in reducing E*. coli* vs. *S. epidermidis* due to catalase activity in the latter being able to break down HP. HPF was equally efficacious in both scenarios. Our third hypothesis was supported with results providing an instance where VA may help reduce TVOCs, as reduction was seen as soon as fogging was completed and persisted throughout the remainder of the study period, remaining below the recommended index threshold of 200.

Except for 48H pre-disinfection for *S. epidermidis*, zero variability existed in CFU counts between readers. Also, except for 48H pre-disinfection for *S. epidermidis*, there was also no variability in CFU counts between plates, i.e., all pre-disinfection plates had 200 CFUs and all post-disinfection plates had zero CFUs. This led to ICC values being undefined. Due to complete agreement between readers, the use of the unanimous CFU as the final value was consistent with the CFU consolidation approach 2. At 48H pre-disinfection for *S. epidermidis*, a single plate contributed to between-reader variability, and two plates contributed to between-plate variability. Although complete between-reader agreement existed for all other plates, the low ICC may have been due to a low degree of variation between plates and may not truly reflect poor reader agreement. Regardless, the CFU consolidation approach 2 was used, which provided the most conservative CFU count.

*E. coli* and *S. epidermidis* were chosen for this study. *S. epidermidis* is a gram-positive, coagulase-negative bacterium and is the most abundant commensal on human skin. It is likely that the more common contaminant on isolation gowns used in medical practice in patient-facing roles isolation gowns are intended for [29]. It is also an accidental pathogen causing opportunistic infections in humans. *S. aureus* (in addition to *E. coli*) is a major source of HAIs, but this organism is a Biosafety level-2 (BSL-2) organism with a moderate potential to cause harm to personnel and the environment [30]. *S. epidermidis* has been proposed as a valuable surrogate for *S aureus* in many applications [31–35]. We thus elected to proceed with *S. epidermidis* in this study as it is a BSL-1, making it safer to handle during and after inoculation.

*E. coli*, also a BSL-1 organism, is a gram-negative rod that is commonly used in disinfection testing as it is an indicator of fecal contamination and is also an organism causing HAIs. It has a thin peptidoglycan cell wall with less coagulase activity than *Staph spp*. Hydrogen peroxide is generally more effective against *E. coli* than *S. epidermidis*, especially in lower concentrations such as those used in this study. *S. epidermidis* with its thick peptidoglycan cell wall that offers protection against reactive oxygen species, as well as catalase positivity that allows for breakdown of hydrogen peroxide to water and oxygen at a higher rate, allowing *Staph spp*, to be less susceptible to hydrogen peroxide when compared to *E. coli* [11,36,37]. While theoretically, this should have made a difference in HP efficacy, our hypothesis was not supported as HP fogging was similarly efficacious.

In a study evaluating the hazards of isolation gowns, *S. epidermidis* was utilized, and found that level 2 isolation gowns, such as those used in this current study, had a 16.3% transmission from the outside to the inside of the gown; this diffusion rate decreased with increasing isolation gown levels [38]. In other studies evaluating the effectiveness of isolation gowns against strikethrough or blood diffusion, gowns were not nearly as effective as we would have thought [39,40]. For this reason, with HPF, we chose to fog both the inside as well as the outside of the gowns, and in addition chose to culture both the inside as well as the outside. We did not separate the cultures of the outside of the gowns from the inside, as other studies have already demonstrated that strikethrough occurs. The total CFU/gown remaining after HPF of both sides of the gown determined the efficacy of whether isolation gowns can be reused.

The McFarland (MF) Standard is a validated method commonly used to ensure consistent inoculum doses in disinfection and antimicrobial susceptibility testing [41,42]. It estimates the concentration of bacterial suspensions based on turbidity when compared to the MF Standard against the background of a Wickerham card. This ranges from 0.5–6.0, and the standards are created from solutions of barium chloride and sulfuric acid that form a precipitate of barium sulfate. The most commonly used standard is 0.5 for disinfection and antimicrobial testing, which is based on spectrophotometer or nephelometer turbidity testing of an approximate bacterial concentration of 1 to 1.5 x 10^8 (CFU/ml); 1.5 x 10^8 is the most widely accepted [43]. While instruments such as a photometric device are used for comparison to the MF standard, visual comparison widely accepted [44] and has been studied both using the naked eye for turbidity comparisons [45] as well as using digital photography with image software analysis of turbidity [46]. In a study evaluating bacterial suspensions of *E. coli* and *S. aureus* by 10 operators when compared to MF 0.5 standard, no statistical difference was found for the mean CFU/ml of *S. aureus* when visually inspected visually vs. a Densicheck photometer [45]. The difference for *E. coli* was statistically different with the eye, resulting in CFU/ml being closer to the standard, more so than the photometer. This further supports objectively that visual inspection can be utilized for the MF standard comparison.

Individual VOCs generated by hydrogen peroxide has been studied in a previous study when household disinfectants were used in the home and office setting [47]. Their published raw data showed that the VOCs generated by 8% hydrogen peroxide were alpha pinene, toluene, trichloromonofluoromethane, xylene m,p, and xylene,o. [48]. When compared to other disinfectants tested, hydrogen peroxide produced the lowest measured TVOC concentrations. However, a recent study suggests that VOCs may not just arise from the disinfectant itself but can also result from the material being disinfected and its interaction with the disinfectant [49]. Polyethylene play mats released the highest concentration of TVOC after disinfection with chlorination, bleaching, UV sterilization, and UV-bleaching disinfection [49]. Isolation gowns are made of polyethylene in addition to polypropylene and may have contributed to the high TVOCs seen in this study following HPF. VOC reduced significantly with the addition of VA remaining below the 200 threshold at all times points with VA suggesting that similar to HPV and HPGP, a post-vacuum cycle may be beneficial with HPF to prevent harm to healthcare workers. Vacuum also helps aid in the drying process by removing moisture from sterilized equipment (post-vacuum).

While the current study suggests vacuum-aeration can limit TVOC generation, constraints in resources prevented replication of VOC measurements beyond the one presented. It is also worth noting the difference in the VOC index and PPM. For example, the maximum VOC index after 90 minutes was 98% of the max VOC index (191) achieved. The maximum PPM after 90 minutes was 84% of the max PPM achieved (0.301). The reason for this discrepancy (98% vs. 84%) may be that the VOC index is a relative adaptive signal while PPM is an absolute concentration. The VOC index is non-linear compared to PPM because both the sensor physics and the Sensirion algorithm are non-linear by design. In other words,the relationship between index and ppm is not fixed. Another possibility is that the methods used to output the values of the VOC index and PPM differ sufficiently to give rise to this discrepancy. If so, it would be important to understand which method is most applicable. It may also be useful to further examine the measurement device to understand whether the range of VOCs being produced due to fogging is compatible with what the device is able to detect.

Limitations of this study are that only Level 2 isolation gowns were used. The level may impact the diffusion of bacteria through the gown, and this may impact the efficacy of HPF. UV radiation could have had some impact in killing both *S epidermidis* and *E. coli*, as both are sensitive to UV radiation, primarily to UV-C [50]. Fogging was done outdoors. UV-C produced by the sun is absorbed by the ozone layer, preventing most (but not all) from reaching the Earth’s surface. Atmospheric conditions could have also impacted disinfection outdoors. Another limitation is that we did not test efficacy against all pathogens commonly encountered in patient-facing roles, such as all bacteria causing hospital-acquired infections, viruses, fungi, and prions. Thus, the efficacy against these pathogens for HPF is unknown. We chose not to separate the cultures from the outside of the gowns from the inside of the gowns. Thus, we do not know specifically if diffusion of microbes occurred from where the gowns were inoculated and how efficacious the disinfection was on each side of the gown as opposed to the gown in general. The use of gowns inoculated with test organisms in this study likely does not replicate complex hospital environments where gowns have the potential to become inoculated with a variety of pathogens during a single use. These results provide some insight into HPF as a viable method of disinfecting isolation gowns. However, isolation gown contamination varies widely in practice. For example, depending on the type of patient facing role (ex., a dermatology, emergency, post-surgical ward) procedure, gowns may receive minimal to extensive exposure to organic material, e.g., blood, soft tissue, skin, infectious material. Further studies be conducted that are more specific to the area of interest and more closely mimic the variety of exposures that would be found before, during, and after those procedures.

In conclusion, our results suggest that HPF offers significant disinfection against common pathogens encountered in HAIs, making this a promising option for disinfection and reuse of isolation gowns. The addition of VA to the process of HPF may help keep TVOCs below the threshold, making it safer for personnel. Further research focusing on using HPF against other pathogens, different levels of isolation gowns, and with gowns naturally inoculated with contaminants during patient-facing roles is needed. Efficacy of using other disinfectants with and without vacuum aeration, such as hypochlorous acid, also needs to be studied.

## Abbreviations

HP: Hydrogen peroxide
HPF: Hydrogen peroxide fogging
VA: Vacuum aeration
HPF-VA: Hydrogen peroxide fogging with vacuum aeration
VOC: Volatile organic compounds
TVOC: Total volatile organic compounds
PPM: Parts per million
BSL: Biosafety level
HAIs: Healthcare acquired infections
MF: McFarland Standard
EO: Ethylene oxide
